# Content validity of the comprehensive home fall hazard checklist, an observational study

**DOI:** 10.1097/MD.0000000000031781

**Published:** 2022-11-25

**Authors:** Christina Ziebart, Neha Dewan, Joy MacDermid

**Affiliations:** a Department of Rehabilitation Sciences, Faculty of Health Science, Western University, London, ON, Canada; b Department of Health Sciences, Lakehead University, Thunder Bay ON, Canada; c Physical Therapy and Surgery, Western University, London, ON, Canada; d Roth|McFarlane Hand and Upper Limb Centre, St. Joseph’s Hospital, London, ON, Canada.

**Keywords:** psychometrics, accidental falls, observational study, checklist

## Abstract

One strategy to reduce the number of falls in older adults is through home hazards assessment checklists. The comprehensive home fall hazard checklist (CHFHC) was designed to guide individuals through their home, assessing fall hazards. The checklist systematically prompts the individuals to check 10 general locations in the house The purpose of this study was to assess the content validity of the comprehensive home fall hazard checklist. A 4-point ordinal Likert rating scale was used to evaluate the content validity of each of the 74 items on the checklist. The relevance and clarity of each item was assessed. Nine experts rated the content validity of each test in relation to the 5 tasks in the rating protocol. The item content validity index, and the scale content validity index were determined, and a kappa rating was calculated. Three of the 74 items on the CHFHC were determined to be not relevant receiving a content validity index of 0.78 or less. All of the items were ranked as being quite clear or highly clear, with all items receiving at least 0.78 on the content validity index. The Kappa score indicates expert agreement. The content validity index was determined to be excellent, with high ratings for both relevance and clarity for 71 of 74 items on the CHFHC.

## 1. Introduction

In North America, an older adult is admitted to emergency from fall related injuries every 13 seconds and dies from a fall every 20 minutes. According to the World Health Organization report on global burden of disease, fall related injuries are the third leading cause of years lived with disability.^[1]^ Furthermore, Nevitt et al^[2]^ reported nearly 57% of seniors experienced a second fall within 1 year. One in ten falls lead to serious injuries, including fractures.^[3]^

One strategy to reduce the number of falls in older adults is through home hazards assessment checklists. Robust evidence from the meta-analysis demonstrates home safety intervention could reduce falls by 39% among at-risk seniors.^[4]^ Home fall-hazard checklists are an easy, useful and effective fall prevention strategy. In a traditional home safety assessment, a therapist tours the home with a fall-hazard checklist to identify potential hazards.^[5–8]^ Therapist home visits to identify and remediate hazards within the home might be considered the gold-standard method for prevention of secondary falls and fractures, but are rarely feasible.^[9,10]^ Currently several falls hazards assessment checklists exist, but with limitations. The Check for Safety: A home Fall Prevention Checklist for Older Adults has 17 items, and is free of cost but currently has no published evidence on usability and effectiveness.^[7]^ The Safe At Home checklist^[11]^ is limited in use to occupational therapists, and cannot be used by patient or caregivers directly. The Safe Living Guide: A guide to Home Safety for Seniors^[12]^ is a comprehensive checklist that addresses 9 different hazard areas such as the outside, inside, stairs, bathroom, kitchen, but is aimed at all populations, not specific for older adults and does not offer solutions for addressing the identified hazards. Finally, the Home Safety Self-Assessment Tool^[13]^ is a reliable and valid tool but is very long and requires an occupational therapist consultation.

Due to these limitations the comprehensive home fall hazard checklist (CHFHC) was developed. The CHFHC checklist was designed to guide individuals through a self-assessment to identify home fall hazards. The checklist systematically prompts the individuals to check 10 general locations in the house: driveway and front door, kitchen, hallways and living room, bedroom, bathroom, stairs, laundry room, garage, general, and apparel. Within each location is a series of prompting questions to observe potential fall hazards. Many of the items are paired with a picture to improve the clarity of the questions. A previous publication described the development process in more detail.^[14]^ The purpose of this study was to assess the content validity of the CHFHC. A secondary purpose was to determine if the validity of the questions changed according to whether it was used by an occupational therapist or a physical therapist.

## 2. Methods

The development of the CHFHC has been previously discussed in another paper.^[14]^ Briefly, the CHFHC is a 74-item checklist, with some questions having subsections, designed for participants to self-administer while walking through their home, identifying factors that could contribute to fall risk. The checklist is divided into 10 categories: driveway and front door, kitchen, hallways and living room, bedroom, bathroom, stairs, laundry room, garage, general, and apparel.

To establish the content validity 9 therapists were invited to complete a content validity index, evaluating both the relevance and clarity of the CHFHC checklist. Therapists were recruited through a mass email was sent out to therapists that have some expertise with falls assessment, and snowballing strategies were used. The amount of time for the therapists to complete the validation was not assessed, however, we gave the therapists 1 month to respond to the email before it was determined that they were unwilling to participate. Response to the email recruitment was considered as consent to participate. Modifications were made based on the feedback of the 9 therapists, and the checklist was re-circulated to 5 of the original therapists and 2 new therapists. These therapists were selected for their expertise in fall hazard identification, and their ability to work with fall-risk populations. In line with the recommendation from Lynn,^[15]^ stating a minimum of 5 and a maximum of ten experts to avoid possible random consensus, this recruited the recommended sample size. All of the experts were from Canada. Four of the experts were physical therapists and 7 of the experts were occupational therapists. Four of the experts (all occupational therapists) hold PhDs. A 4-point ordinal Likert rating scale was used to evaluate the content validity of each of the 74 items on the checklist. The relevance and clarity of each item was assessed. The 16 experts rated the content validity of each test in relation to the 5 tasks in the rating protocol. The scale was scored accordingly: 1 = test not being relevant; 2 = somewhat relevant; 3 = quite relevant and; 4 = highly relevant. Grades 3 and 4 were considered acceptable.^[15]^ Results from the 11 experts´ judgements were quantified. This study received ethics approval from the McMaster Research Ethics Board.

Statistical analyses were conducted using Microsoft Excel version 16.38. (Microsoft 365, Redmond, Washington). For establishing content validity, the content validity index (CVI) was calculated by dividing the number of experts that arrived at an accepted test grade 3 (quite relevant) or 4 (highly relevant) by the total number of assessments of the test.^[15,16]^ The cutoff for an excellent level was > 0.78 for the tests.^[16]^

A modified kappa value was calculated, the Kappa designating agreement of relevance, using the formula: (k = (CVI − Pc)/(1 − Pc).^[16,17]^ The probability of chance occurrence (Pc) was computed using the formula for a binominal random variable, with 1 specific outcome: Pc = [N!/A! (N–A)!].5N where N = number of experts and A = Number agreeing on good relevance.^[18]^ The component of the formula “!” is a mathematical symbol for the product of all positive interfere less than or equal to N, for example 5! to mean 5 × 4 × 3 × 2 × 1.^[17]^ Guidelines to evaluate the relevance of the tests were applied using an evaluation criterion for considering values for kappa, as proposed by Cicchetti and Sparrow: Fair = k_of 0.40 to 0.59; Good = k_ of 0.60 to 0.74; and Excellent = k_ of 0.75 to.^[17,19]^ A score of 1 was given to those items rated as 3 or 4 and a score of 0 was given to those items rated 2 or 1. All the scores were added together to give a total out of 97, accounting for sub-components of certain questions. A score of 97 would indicate that all items were determined to have high relevance or high clarity. An acceptable score is 80%.^[17,19]^

Finally, scale-level content validity was calculated in 2 different ways. Firstly, the proportion of items on the scale that achieves a relevance rating of 3 or 4 by all the experts was calculated at the scale (S)-CVI/ universal agreement (UA). The number of items rated as 3 or 4 by all the experts were then divided by the total number of items (97). The S-CVI/UA was then broken down by therapists, so then all the physical therapists rating the item as highly relevant or clear was determined and divided by the total number of items; similarly for the occupational therapists, all the items that were rated as 3 or 4 were determined and divided by the total number of items on the checklist. Secondly, the average of each item was calculated for all the items on the scale, which is represented as the S-CVI/Avg. The S-CVI/Avg summed all the item content validity index scores and divided by the total number of items, which was 97 (after accounting for sub-components of questions). When looking at the validity of the overall scale, the S-CVI/UA is determined to be acceptable at a level of 0.4.^[18,20]^

## 3. Results

### 3.1. Item level CVI

Items numbered 11, 21, and 72 were identified as being less relevant due to scoring less than 0.78 on the on the CVI. These items are: “can you easily and comfortably step through the entrance/ threshold to your home;” “do you have access to a telephone on each floor of your house;” and “do you have a mat right beside your door to wipe your feet.” The item related to having a telephone on each floor was ranked the least relevant, receiving a CVI score of 0.44 (Table 1).

**Table 1:** Mean item level content validity index. The I-CVI is calculated by the number of experts rating the item as a 3 or 4. A score of 100% indicates all experts agreed that the item was relevant by rating is a 3 or 4. A score of 78% or higher was deemed acceptable. Three items on the total number of experts were determined have a score of less than 78% of the experts rating the item as relevant. All items were determined as clear.

Item number	Total		PT		OT		Kappa			
	Mean I-CVI total relevance	Mean I-CVI Total clarity	Mean I-CVI PT relevance	Mean I-CVI PT clarity	Mean I-CVI OT relevance	Mean I-CVI OT clarity	Kappa relevance	Rating	Kappa clarity	Rating
1	1.00	1.00	1.00	1.00	1.00	1.00	1.00	Excellent	1.00	Excellent
2	1.00	0.89	1.00	0.75	1.00	1.00	1.00	Excellent	0.89	Excellent
3	1.00	1.00	1.00	1.00	1.00	1.00	1.00	Excellent	1.00	Excellent
4	1.00	1.00	1.00	1.00	1.00	1.00	1.00	Excellent	1.00	Excellent
5	1.00	0.89	1.00	1.00	1.00	0.80	1.00	Excellent	0.89	Excellent
6	0.89	0.89	0.75	1.00	1.00	0.80	0.89	Excellent	0.89	Excellent
7	1.00	1.00	1.00	1.00	1.00	1.00	1.00	Excellent	1.00	Excellent
8	0.89	0.89	1.00	1.00	0.80	0.80	0.89	Excellent	0.89	Excellent
9	1.00	1.00	1.00	1.00	1.00	1.00	1.00	Excellent	1.00	Excellent
10	1.00	0.89	1.00	1.00	1.00	0.80	1.00	Excellent	0.89	Excellent
11	0.67	0.89	0.50	1.00	0.80	0.80	0.66	Good	0.89	Excellent
12	0.89	0.78	0.75	1.00	1.00	0.60	0.89	Excellent	0.77	Excellent
13	0.89	0.89	1.00	0.75	0.80	1.00	0.89	Excellent	0.89	Excellent
14	0.78	0.89	0.50	1.00	1.00	0.80	0.77	Excellent	0.89	Excellent
15	1.00	1.00	1.00	1.00	1.00	1.00	1.00	Excellent	1.00	Excellent
16	1.00	0.89	1.00	0.75	1.00	1.00	1.00	Excellent	0.89	Excellent
17	1.00	1.00	1.00	1.00	1.00	1.00	1.00	Excellent	1.00	Excellent
18	1.00	0.78	1.00	0.75	1.00	0.80	1.00	Excellent	0.77	Excellent
19	1.00	0.78	1.00	0.75	1.00	0.80	1.00	Excellent	0.77	Excellent
20	0.78	1.00	1.00	1.00	0.60	1.00	0.77	Excellent	1.00	Excellent
21	0.44	1.00	0.50	1.00	0.40	1.00	0.42	Fair	1.00	Excellent
22	0.89	1.00	1.00	1.00	0.80	1.00	0.89	Excellent	1.00	Excellent
23	1.00	0.89	1.00	0.75	1.00	1.00	1.00	Excellent	0.89	Excellent
24	1.00	1.00	1.00	1.00	1.00	1.00	1.00	Excellent	1.00	Excellent
25	1.00	0.89	1.00	0.75	1.00	1.00	1.00	Excellent	0.89	Excellent
26	1.00	0.89	1.00	0.75	1.00	1.00	1.00	Excellent	0.89	Excellent
27	0.89	1.00	1.00	1.00	0.80	1.00	0.89	Excellent	1.00	Excellent
28	0.89	0.89	0.75	0.75	1.00	1.00	0.89	Excellent	0.89	Excellent
29	0.89	1.00	1.00	1.00	0.80	1.00	0.89	Excellent	1.00	Excellent
30	0.89	1.00	0.75	1.00	1.00	1.00	0.89	Excellent	1.00	Excellent
31	1.00	0.78	1.00	0.75	1.00	0.80	1.00	Excellent	0.77	Excellent
32	0.89	1.00	1.00	1.00	0.80	1.00	0.89	Excellent	1.00	Excellent
33	1.00	1.00	1.00	1.00	1.00	1.00	1.00	Excellent	1.00	Excellent
34	0.89	1.00	0.75	1.00	1.00	1.00	0.89	Excellent	1.00	Excellent
35	1.00	1.00	1.00	1.00	1.00	1.00	1.00	Excellent	1.00	Excellent
36	1.00	1.00	1.00	1.00	1.00	1.00	1.00	Excellent	1.00	Excellent
37	1.00	0.89	1.00	0.75	1.00	1.00	1.00	Excellent	0.89	Excellent
38	1.00	1.00	1.00	1.00	1.00	1.00	1.00	Excellent	1.00	Excellent
39	1.00	1.00	1.00	1.00	1.00	1.00	1.00	Excellent	1.00	Excellent
40	1.00	0.89	1.00	1.00	1.00	0.80	1.00	Excellent	0.89	Excellent
41	0.78	0.89	**0.50**	0.75	1.00	1.00	0.77	Excellent	0.89	Excellent
42	0.89	1.00	0.75	1.00	1.00	1.00	0.89	Excellent	1.00	Excellent
43	0.89	0.89	0.75	0.75	1.00	1.00	0.89	Excellent	0.89	Excellent
44	1.00	1.00	1.00	1.00	1.00	1.00	1.00	Excellent	1.00	Excellent
45	0.89	1.00	1.00	1.00	0.80	1.00	0.89	Excellent	1.00	Excellent
46	1.00	1.00	1.00	1.00	1.00	1.00	1.00	Excellent	1.00	Excellent
47	1.00	0.78	1.00	1.00	1.00	0.60	1.00	Excellent	0.77	Excellent
48	0.89	0.78	0.75	0.75	1.00	0.80	0.89	Excellent	0.77	Excellent
49	0.89	0.78	0.75		1.00	0.80	0.89	Excellent	0.77	Excellent
50	1.00	1.00	1.00	1.00	1.00	1.00	1.00	Excellent	1.00	Excellent
51	1.00	1.00	1.00	1.00	1.00	1.00	1.00	Excellent	1.00	Excellent
52	1.00	0.89	1.00	1.00	1.00	0.80	1.00	Excellent	0.89	Excellent
53	0.89	1.00	1.00	1.00	0.80	1.00	0.89	Excellent	1.00	Excellent
54	1.00	1.00	1.00	1.00	1.00	1.00	1.00	Excellent	1.00	Excellent
55	1.00	1.00	1.00	1.00	1.00	1.00	1.00	Excellent	1.00	Excellent
56	1.00	1.00	1.00	1.00	1.00	1.00	1.00	Excellent	1.00	Excellent
57	1.00	1.00	1.00	1.00	1.00	1.00	1.00	Excellent	1.00	Excellent
58	1.00	1.00	1.00	1.00	1.00	1.00	1.00	Excellent	1.00	Excellent
59	0.89	1.00	0.75	1.00	1.00	1.00	0.89	Excellent	1.00	Excellent
60	1.00	1.00	1.00	1.00	1.00	1.00	1.00	Excellent	1.00	Excellent
61	1.00	1.00	1.00	1.00	1.00	1.00	1.00	Excellent	1.00	Excellent
62	1.00	1.00	1.00	1.00	1.00	1.00	1.00	Excellent	1.00	Excellent
63	0.89	0.89	0.75	0.75	1.00	1.00	0.89	Excellent	0.89	Excellent
64	1.00	1.00	1.00	1.00	1.00	1.00	1.00	Excellent	1.00	Excellent
65	1.00	0.89	1.00	1.00	1.00	0.80	1.00	Excellent	0.89	Excellent
66	1.00	1.00	1.00	1.00	1.00	1.00	1.00	Excellent	1.00	Excellent
67	1.00	1.00	1.00	1.00	1.00	1.00	1.00	Excellent	1.00	Excellent
68	0.89	1.00	0.75	1.00	1.00	1.00	0.89	Excellent	1.00	Excellent
69	0.89	0.78	0.75	0.75	1.00	0.80	0.89	Excellent	0.77	Excellent
70	0.89	0.89	1.00	1.00	0.80	0.80	0.89	Excellent	0.89	Excellent
71	1.00	0.89	1.00	1.00	1.00	0.80	1.00	Excellent	0.89	Excellent
72	0.67	0.89	0.75	1.00	0.60	0.80	0.66	Good	0.89	Excellent
73	0.89	1.00	0.75	1.00	1.00	1.00	0.89	Excellent	1.00	Excellent
74	1.00	1.00	1.00	1.00	1.00	1.00	1.00	Excellent	1.00	Excellent

I-CVI = item content validity index.

All of the questions were ranked as being quite clear or highly clear, with all items receiving at least 0.78 on the CVI (Table 1).

As a result of some of the items being rated as not clear, they were modified to reflect the suggestions from the therapists. Item 11 changed from: “can you easily and comfortably step through the entrance/ threshold to your home?” to “is it easy and safe for you to step through the entrance/ threshold of your house?.” Item 21 changed from “do you have access to a telephone on each floor of your house” to “do you have access to telephone on the floor(s) where you live?.” Item 72 was changed from “do you have a mat right beside your door to wipe your feet” to “do you have a secure mat right beside your door(s) to wipe your shoes/feet?.”

Seven of the therapists were asked to reevaluate the checklist with the modifications. In the revised version 3 new items received a rating indicating the question was not relevant, but all items were determined to be clear. In this version, items 4, 21 and 41 were determined to be not relevant. These items were: item 4- “Is there enough space to enter and exit your car safely?”; item 21- “do you have a bathroom on each floor (s) of your house?”; item 41- “What type of bathroom floor do you have?.”

### 3.2. Modified kappa value

The kappa score indicates expert agreement. The agreement was generally excellent among all of the items. Two items received a rating of good, and 1 item received a rating of fair. The 2 “good” items were items 11 “can you easily and comfortably step through the entrance/threshold to your home;” and item 72 “do you have a mat right beside your door to wipe your feet.” Item 21 received a fair agreement which was the question: “do you have access to a telephone on each floor of your house” (Table 1).

In the second iteration of evaluation of the checklist items 4, 21 and 41 received a kappa value of “good,” otherwise all items received a kappa value of excellent for relevance. Item 22 received a kappa of “good” for clear and all other items received a kappa value of excellent.

### 3.3. CVI differences based on discipline

We evaluated whether physical therapists viewed the items as having a different level of relevance or clarity compared to the overall rating and compared to the rating of occupational therapists, recognizing that our numbers were very small and may not be representative of these groups. For the physical therapists a score of 0.75 or higher was deemed relevant or clear, meaning that 3 of the 4 therapists agree, which well exceeded the benchmark of acceptability which was set as 0.4. Items number 11, 14, 21, and 41 were deemed to be not relevant by physical therapists. These items are: “can you easily and comfortably step through the entrance/threshold to your home;” “is there enough counter space in your kitchen;” “do you have access to a telephone on each floor of your house;” and “are there raised edges on the stair treads.” Items 14 and 41 were uniquely identified as not relevant by the physical therapists. All items were ranked as being clear, receiving a CVI of 0.75 or greater (Table 1).

The occupational therapists identified items numbered 20, 21, and 72 as not relevant. These items are: “do you have a bathroom on each floor of your house;” “do you have access to telephone on each floor of your house;” and “do you have a mat right beside your door to wipe your feet.” Item 20 was identified as not relevant by occupational therapists, but not physical therapists. Two items were ranked as not clear, receiving a score of 60%. Item 12: “in your kitchen, do you keep the items you use most on shelves between your shoulder and hip level” and item 47: “are the steps a reasonable steepness? Is the tread about 279 mm (11”) and is the riser about 178 mm (7”)” were assessed as unclear by the occupational therapists (Table 1).

Only item 21 was consistently reported as not relevant across both health care professionals. Table 2 shows the content validity of the overall scale showing the rating provided by each therapist for both relevance and clarity of the checklist.

**Table 2:** Content validity of overall scale, with the results of each therapists rating. A score of 1 was given to those items rated as 3 or 4 and a score of 0 was given to those items rated 2 or 1. All the scores were added together to give a total out of 97, accounting for sub-components of certain questions. A score of 97 would indicate that all items were determined to have high relevance or high clarity. An acceptable score is 80%.

Therapist	Relevance	Proportion relevant	Clarity	Proportion clarity
1 PT	78	0.80	89	0.91
2 PT	97	1	97	1
3 PT	95	0.97	94	0.97
4 PT	93	0.96	91	0.94
5 OT	95	0.98	77	0.79
6 OT	91	0.94	82	0.84
7 OT	94	0.97	94	0.97
8 OT	94	0.96	95	0.98
9 OT	91	0.94	95	0.98
10 OT	97	1	97	1
11 OT	92	0.95	89	0.92

OT = occupational therapist, PT = physical therapist.

### 3.4. S-CVI/UA

The S-CVI/UA is an overall scale rating, indicating the proportion of therapists that rated all items as a 3 or 4. For relevance the score was 0.61 and for clarity the score was 0.58. The cut off score to be acceptable was set at 0.4, suggesting these scores are clear and relevant. When looking at the therapist level rating, the proportion physical therapists that ranked all items as 3 or 4 for relevance was 0.74 and for clarity was 0.77. When looking at the occupational therapists, the proportion that ranked all items as 3 or 4 was 0.82 for relevance and 0.73 for clarity (Table 3).

**Table 3:** Content validity of overall scale. The S-CVI/Avg summed all the I-CVI scores and divided by the total number of items, which was 74. the proportion of items on the scale that achieves a relevance rating of 3 or 4 by all of the experts was calculated at the S-CVI/UA, which was then divided by the total number of items on the checklist.

	S-CVI/Avg	S CVI-UA
Total relevance	0.94	0.61
Total clarity	0.94	0.58
PT relevance	0.92	0.74
PT clarity	0.94	0.77
OT relevance	0.95	0.82
OT clarity	0.94	0.73

I-CVI = item content validity index, OT = occupational therapist, PT = physical therapist, S-CVI = scale level content validity, UA = universal agreement.

### 3.5. S-CVI/Avg

The S-CVI is again an overall scale statistic indicating the average score of all the items. This provides an average score of all the items on the scale. A score of 3 or 4 was coded as 1 and a score of 2 or 1 was coded as zero, providing an indication of the item being relevant/clear or not relevant/clear. The overall score was provided for each therapist with a score 74 being the highest, and then divided by 74 for provide the average score. Overall, for all therapists, the relevance received an S-CVI/Avg of 94% and the clarity received an S-CVI/Avg of 94%. Physical therapists provided a score of 92% for relevance and 94% for clarity. Occupational Therapists provided a relevance score of 95% and a clarity score of 94% (Table 3).

## 4. Discussion

The main finding was that 71 of 74 items were considered content valid and relevant for fall hazard detection on the CHFHC. All of the items were interpreted as being clear by the evaluators. Of the items that were not considered valid, it was due to their relevance. When looking at the validity of the overall scale, the S-CVI/UA is determined to be acceptable at a level of 0.4, the relevance and clarity of the overall scale, as well as the relevance and clarity rated by physical and occupational therapists all exceed the 0.4 cut off. Similarly, the S-CVI/Avg is acceptable at 0.9, which is achieved in relevance and clarity. Overall, it is determined that most of the items on the CHFHC have content validity, and the overall scale is content valid.

The item that was rated the lowest and had to be modified was related to having a telephone on every floor of the house. It was likely that this item was rated as not relevant due to the increased use of cellular telephones, allowing for portable and constant access to a phone. Currently more than 50% of older adults are using cellphones,^[21]^ suggesting that this question may not be relevant in the questionnaire and that cellphone are widely adapted by older adults. However, with the increased use of cellphones in older adults, consideration needs to be given towards the use of the cellphone while walking, which can increase fall risk. The use of the cellphone while walking is a dual-task,^[22]^ which can increase the cost of performance, leading to worse performance on tasks, such as crossing the street,^[23]^ or increasing the risk of falling. The use of cellphones in the house may need to be considered in future iterations of fall hazards checklists to confirm that the client is not using their phone while walking or moving about the house.

There were some discrepancies in the item ratings across the therapists, with the occupational therapists finding 3 items not relevant and 2 items not clear, and the physical therapists identifying 4 different items as not relevant and none of the items as unclear. These findings suggest that there may be vocational differences in the interpretations of the checklists. It is very important to consider the perspective of both occupational and physical therapists, as both professions may be administering fall hazards checklists to the clients. However, we also recognize that there were very small subgroups, and this just may be chance differences not based on occupational differences.

When judging the overall content validity of the scale we found that the content validity was very high and met the cut off points suggested by Lynn.^[15]^ The S-CVI/UA is an indication of the proportion of therapists raking all the items on the checklist as relevant and clear and we achieve a score of 0.68, suggesting that the checklist is almost entirely relevant and clear. When looking at the average score of the checklist is was 0.98 again, indicating that on average all the therapists rated the checklist as relevant and clear. When comparing to other checklists, the CHFHC has among the highest content validity index.^[24]^ The Westmead Home Safety Assessment had a content validity index of 0.8,^[25]^ the COUGAR home safety assessment had a content validity of an acceptable level,^[26]^ the Housing Enabler checklist was determined to be a satisfactory level,^[27]^ and finally the Home Fast self-assessment tool was also determined to have satisfactory content validity.^[28]^ Despite the excellent content validity of the CHFHC, further psychometric properties need to be explored.

When deciding whether to delete, revise or keep an item number of factors should be considered. Content validity indices can help identify items that may be problematic, but deletion of items without consideration for alternatives is not necessarily the best strategy. Patient burden, theoretical importance, and whether an item might be critically important but to a small percentage of people are all factors that should be considered. Although the questionnaire is very long, unlike outcome measures which are administered heatedly the intention of this home hazard assessment is that it would only need to be completed on a single occasion. In fact, if participants wish to chunk the assessment into smaller multiple time frames that would be perfectly acceptable given that the intention is to identify home hazards not to assess patient status at a specific point in time. Therefore, some of the rules that might be used for deleting items in outcome measures may not apply to this type of tool. Conversely, deleting an item that is a non-common but highly hazardous factor that has theoretical relationship to falls might not be the best strategy even though the content validity index indicates problems. Rather problems may sometimes be related to the fact that the item is unclear. For example, we think that the telephone item is outdated given changes in how people use portable telephones, rather than land lines which were confined to a specific space. Concerns about the content validity about items around a mat at the door might relate to that entranceway mat could be preventative if it is secure and absorbs water to reduce slipperiness upon entry of the home but could be a fall hazard it is loose or a tripping obstacle. In this case the item could be rehabilitated by simply specifying that the mat would have to be secure. Finally, it must be remembered that expert evaluation of the items would be placed in context of all the other proposed items and given that so many of the item’s word deemed highly relevant, raters might feel that part of their job is to identify some items is not being relevant. Since this was a quantitative method not a qualitative study, we could not explore the reasons for their choices.

We believe that this questionnaire is a useful tool to integrate into clinical practice as is provides a comprehensive evaluation of potential fall hazards. It could be used for patients/clients that are recurrent fallers. This checklist can be used as an evaluation of potential hazards and then allow therapists to provide education and intervention on how to reduce the risk of falls. Further, the tool could be used to evaluate the efficacy of fall hazard interventions in research. Future research should further assess the psychometric properties of this checklist, and then use the checklist in an intervention to reduce fall hazards, and then determine if the reduction of fall hazards leads to a reduction in falls.

## 5. Strengths and limitations

This study focused on a quantitative assessment of content validity of the CHFHC. Content validity can also be examined by cognitive interviews and content classification for example, ICF linking. Other psychometric properties should be evaluated, including the face validity. Since this is a screening questionnaire to identify fall hazards it is appropriate that interrater reliability be examined and criterion validity be examined by comparing self-assessment by an individual patient, family member or support personnel in comparison to a gold standard fall assessment home evaluation performed by professional. The extent to which such assessments help with risk modification should be evaluated. For the current study we did modify several items to improve clarity. Table 4 shows the original question next to the modified question. At this stage, none of the questions were removed. We felt that although 3 of the questions were determined to not be relevant, removing them would leave a large gap in the evaluation of home fall hazards assessment. For example, removing the question about accessing the phone would leave a gap of knowledge related to whether the patient had access to a phone to call for help if they fell. The updated version of the CHFHC can be found in Appendix A.

**Table 4:** A comparison of the original and modified questions that were determined to be not clear or not relevant, and how they have been modified in the recent iteration of the comprehensive home fall hazard checklist. The modified components are bolded.

Original question	Modified question
Can you easily enter your home? Are you free from any entrance problems (e.g., unlocking the door; in an apartment, maneuvering from entering passcode to the door; getting buzzed in and rushing to the door)?	Can you easily **and safely** enter your home? Are you free from any entrance problems (e.g., unlocking the door; in an apartment, maneuvering from entering passcode to the door; getting buzzed in and rushing to the door)?
Is there any step or threshold at the entrance of your home?	**Can you easily and safely step through the entrance/threshold to your home?**
Can you reach most objects in your cupboard from a standing height?	**Can you safely and comfortably reach most objects in your cupboard from a standing height?**
Do you have a bathroom on each floor of your house?	Do you have a bathroom on each **floor (s**) of your house?
Do you have a telephone on each floor of your house?	Do you have a telephone the floor **(s) where you live?**
Are the steps a reasonable steepness? Is the tread about 279 mm (11”) and is the riser about 178 mm (7”)	**Is the riser of each step a comfortable standard (about 7ʺ or 18 cm)?**
Do you have a mat right beside your door to wipe your feet?	Do you have a **secure** mat right beside your door (s) to wipe your feet?

## 6. Conclusion

In conclusions, this study evaluated the content validity of the CHFHC. The content validity index was determined to be excellent. Three items did not meet the cutoff for being relevant, when assessed by all 9 therapists. One item was unanimously determined to be not-relevant. Overall, the CHFHC is a relevant and clearly designed checklist.

## Author contributions

**Conceptualization:** Neha Dewan, Joy MacDermid.

**Formal analysis:** Christina Ziebart.

**Funding acquisition:** Neha Dewan, Joy MacDermid.

**Investigation:** Christina Ziebart, Joy MacDermid.

**Methodology:** Christina Ziebart, Neha Dewan, Joy MacDermid.

**Resources:** Joy MacDermid.

**Supervision:** Joy MacDermid.

**Validation:** Christina Ziebart.

**Writing – original draft:** Christina Ziebart.

**Writing – review & editing:** Christina Ziebart, Neha Dewan, Joy MacDermid.
